# Comparison of *in vitro* antioxidant activities of kefir, yogurt, and cheese produced from goat milk

**DOI:** 10.1016/j.fochx.2025.103394

**Published:** 2025-12-09

**Authors:** Hasan Uzkuç, Sümeyye Sarıtaş, Nesrin Merve Çelebi Uzkuç, Yonca Karagül Yüceer, Tuba Esatbeyoglu

**Affiliations:** a Department of Molecular Biology and Genetics, Faculty of Science, Çanakkale Onsekiz Mart University, Terzioglu Campus, 17020, Çanakkale, Türkiye; b Department of Food Engineering, Faculty of Engineering, Çanakkale Onsekiz Mart University, Terzioglu Campus, 17020, Çanakkale, Türkiye; c Department of Molecular Food Chemistry and Food Development, Institute of Food and One Health, Gottfried Wilhelm Leibniz University Hannover, Am Kleinen Felde 30, 30167 Hannover, Germany

**Keywords:** Goat milk, Fermentation, Bioaccessibility, *In vitro*, CUPRAC, FRAP, Bioactive peptide

## Abstract

This study compared the physicochemical characteristics and *in vitro* antioxidant activities of kefir, yogurt, and cheese produced from the same batch of goat milk using specific starter cultures. The physicochemical properties of the samples, as well as the antioxidant capacity (CUPRAC and FRAP) of the *in vitro* digested samples were analyzed. Additionally, low molecular weight (<3 kDa) peptide fractions were isolated to evaluate their contribution to antioxidant capacity. The highest antioxidant activities (mM Trolox/g protein) based on the CUPRAC assay were observed in yogurt (123.30), followed by kefir (116.05) and cheese (89.35). In contrast, the highest FRAP values were in cheese (22.74), followed by kefir (14.22) and yogurt (14.12). Low molecular weight (<3 kDa) peptide fractions generally showed antioxidant activity similar to or higher than that of the corresponding complete digest samples. Findings highlight the antioxidant potential of fermented goat milk products, especially cheese and kefir, for functional dairy applications.

## Highlights

• Comparison of the antioxidant capacity of goat milk kefir, yogurt, and cheese
• <3 kDa fractions showed strong antioxidant potential after digestion
• Findings support the functional food potential of goat milk products

## 1 Introduction

In recent years, interest in healthy eating and living has rapidly increased, especially after the Covid-19 pandemic (Sarıtaş, Mondragon Portocarrero, Miranda, Witkowska, & Karav, 2024). Consequently, the importance of food products with health-promoting properties has grown. The Food and Agriculture Organization of the United Nations (FAO), in the guide prepared in the coordination of the World Health Organization (WHO), recommends daily consumption of milk and dairy products (such as cheese, yogurt, kefir) due to their high nutritional value and beneficial health effects on the human body (FAO, 2020). Dairy products contain various antioxidant compounds including proteins (especially casein), peptides, antioxidant enzymes (superoxide dismutase (SOD), catalase and glutathione peroxidase), conjugated linoleic acid (CLA), coenzyme Q10, lactoferrin, vitamins (C, E, A, and D3), carotenoids, some minerals and some trace elements. The content of these compounds varies depending on the product matrix (milk, yogurt, fermented milk and cheese) and processing methods (mechanical, thermal and fermentative) (Grażyna, Hanna, Adam, & Magdalena, 2017; Stobiecka, Król, & Brodziak, 2022).

The source of milk, depending on the species and breed of the animal, is critically important for determining the antioxidant compounds present in the final product. Among milk types, goat, cow, and donkey milk are notable for their richness in bioactive components and their higher antioxidant potential (Simos et al., 2011). The smaller fat globules in goat milk allow it to remain in suspension longer than cow milk, reducing the need for homogenization. Short and medium chain fatty acids in goat milk contribute to its easier digestibility (Lad, Aparnathi, Mehta, & Velpula, 2017). It has been reported to be preferable to cow milk due to its easy digestibility, strong buffering capacity, alkalinity and potential therapeutic applications (Lad et al., 2017). Furthermore, it is considered more suitable than cow milk for individuals with lactose intolerance due to its lower lactose content (Filipović Dermit, 2014). Taurine in goat milk has been reported to reduce the effects of cardiovascular disease and hypertension (Thakur et al., 2024). Thanks to its richer mineral and vitamin content compared to cow milk, goat milk exhibits greater bioavailability in the human intestine. Therefore, goat milk and its derived products may help meet certain nutritional requirements in the human diet (Saikia, Hassan, & Walia, 2022). In addition to its nutritional value, goat milk demonstrates nutraceutical properties, rendering it suitable for infants, the elderly, and patients undergoing treatment (Verma et al., 2025).

Among milk-based fermented foods, kefir, yogurt, and cheese are widely consumed thanks to their high nutritional value (Bazán Tantaleán, Del-Río, Cortés Diéguez, Domínguez, & Pérez Guerra, 2024; Sarıtaş, Mondragon Portocarrero, et al., 2024). These products are rich sources of protein, calcium, vitamins and beneficial microorganisms (Sarıtaş, Mondragon Portocarrero, et al., 2024). Yogurt and kefir, especially, support intestinal health through their diverse probiotic content (Bazán Tantaleán et al., 2024). Cheese, on the other hand, serves as a concentrated source of protein and minerals, with a longer shelf life and a rich nutritional profile (Barac et al., 2016). Dairy products promote digestive health, bone health and development, and immune system function.

Antioxidant ingredients are used for therapeutic purposes to prevent possible damage caused by free radicals, and research is being conducted to promote the antioxidant content of various food products (Sarıtaş et al., 2024). Given the widespread daily consumption of milk and dairy products and their notable antioxidant properties, their potential as a natural supplement is under investigation (Xiong, 2010). Due to the potential carcinogenic effects of synthetic antioxidants, there is growing interest in natural antioxidants; however this has also led to uncertainty regarding their efficacy and safety (Liu, Lin, Chen, Chen, & Lin, 2005). Meanwhile, the use of amino acids, peptides and proteins as food antioxidants is gaining popularity due to their low cost, safety and high nutritional value (Park, Morimae, Matsumura, Nakamura, & Sato, 2008).

The antioxidant activity of proteins is largely attributed to specific amino acids in their structures and bioactive peptides (5–11 amino acids) generated through enzymatic hydrolysis by proteases. For instance, hydrophobic amino acids such as proline, histidine, tyrosine and tryptophan along with their derivatives, have demonstrated antioxidant properties. Antioxidant peptides have been successfully isolated from milk casein and whey proteins in multiple studies (Hernández-Ledesma, Dávalos, Bartolomé, & Amigo, 2005; Suetsuna, Ukeda, & Ochi, 2000). These peptides have been shown to inhibit both enzymatic and non-enzymatic peroxidation of essential fatty acids, with majority derived from the α-casein sequence (Fitzgerald & Murray, 2006). A study reported that casein hydrolysates exhibit scavenging activity on superoxide anion (O_2_^−^), hydroxyl radical, and 1,1-diphenyl-2-picrylhydrazyl (DPPH) radicals Suetsuna et al. (2000). The glutamine-leucine sequence from casein was identified as critical for the radical scavenging activity, suggesting that the primary protein structure significantly influences antioxidant function. The proteolytic activity of the probiotic *Lactobacillus fermentum* strain, when used alongside starter cultures in cheese production, released a large number of casein-derived peptides and significantly enhanced the antioxidant activity of probiotic cheeses during storage compared to those made solely with starter cultures (Songisepp et al., 2004).

Bioactive peptides in products such as cheese, yogurt and kefir obtained from goat milk exhibit antioxidant properties (Barac et al., 2016; Moatsou & Park, 2017). A study reported that the total antioxidant capacities of water-soluble and water-insoluble protein fractions increased after 50 days of storage in brine cheese prepared by heat treating goat milk at 90 °C for 10 min (Barac et al., 2016).

Goat milk goes through different processes during its use for the production of different products. These processes cause changes in the components of goat milk, especially in its bioactive content. However, in fermented milk products, bioactive peptides are released from the protein chain by digestive enzymes, proteolytic microorganisms and microorganism-derived enzymes. This process depends on the milk and the starter culture used. Although various studies have been conducted on the bioactive peptides of kefir, yogurt and cheese produced from goat milk, there are no studies comparing the antioxidant activities of these products after *in vitro* digestion. In this study, the *in vitro* antioxidant activities of post pancreatic and low molecular weight (<3 kDa) fractions of kefir, yogurt, and cheese produced from the goat milk were compared.

## 2 Materials and methods

### 2.1 Material

Daily goat's milk (İmbroz Co. Inc., Çanakkale, Türkiye) was used in the production of kefir, yogurt and cheese. The pH value and gross chemical composition of the goat milk were 6.49 pH, 12.18% dry matter (DM), 4.37% total protein, and 4. 26% fat content. Commercial starter cultures specifically produced for each product were supplied by Chr. Hansen (Hørsholm, Denmark). For kefir production, a mesophilic/thermophilic kefir culture (Exact® Kefir 1) containing *Debaryomyces hansenii, Lactococcus lactis* ssp. *cremoris, Lactococcus lactis* ssp. *lactis, Lactococcus lactis* ssp. *lactis biovar diacetylactis, S. thermophilus* and *Leuconostoc* was used; for yogurt production, a thermophilic yogurt culture (Yoflex® YF-L812) containing *Lactobacillus delbrueckii* ssp. *bulgaricus* and *Streptococcus thermophilus* was used; and for cheese production, a mesophilic homofermentative cheese culture (R707) containing *Lc. lactis* ssp. *lactis* and *Lc. lactis* ssp. *cremoris* was used. A calf rennet (Rumeli Maya, İstanbul, Türkiye) which had 180 IMCU/mL value was used for coagulation of cheese milk. The enzymes used in the *in vitro* digestion experiment were pepsin from porcine gastric mucosa (P7012), pancreatin from porcine pancreas 8 × USP (P7545), and porcine bile extract (B8631), all of which were purchased from Sigma-Aldrich (St. Louis, MO, USA).

### 2.2 Production of kefir, yogurt and cheese

**Fig. 1:**
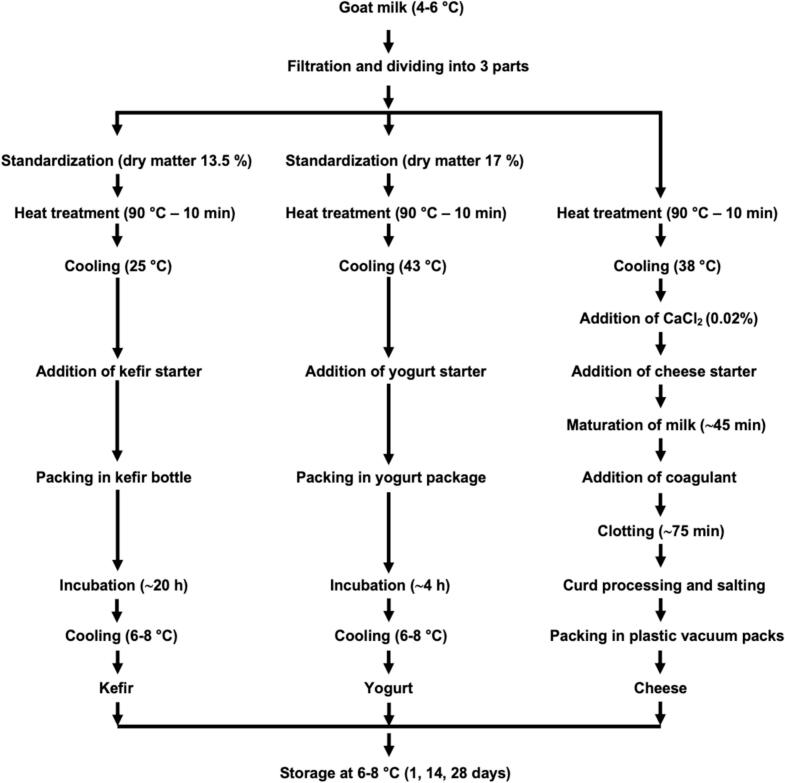
Production flowsheet of kefir, yogurt and cheese.

Experimental kefir, yogurt and cheese were manufactured in a dairy plant (Ekozey Co. Inc., Çanakkale, Türkiye). Goat milk was divided into three parts for production. Each product was manufactured using process parameters suitable for it (Fig. 1). Evaporation process was applied for kefir and yogurt milk. DM of kefir milk was standardized to 13.5%, and the DM of yogurt milk was standardized to 17%. Kefir, yogurt and cheese milk were heat-treated at 90.0 ± 1 °C for 10 min and cooled to the fermentation temperature for each product. Starter cultures used in kefir, yogurt, and cheese production were added in the amounts specified in the product information sheet to initiate fermentation. All productions were carried out in two replicates.

#### 2.2.1 Kefir production

Heat-treated goat milk was cooled to 25.0 ± 1 °C and 0.1 U/L kefir starter culture was added. The fermented milk was filled into 1-L glass kefir bottles and incubated for approximately 20 h, with periodic mixing until the pH reached 4.6. The kefir, which reached the target pH level, was quickly cooled and kept in the refrigerator at 6–8 °C for one day before being analyzed.

#### 2.2.2 Yogurt production

Heat-treated goat milk was cooled to 43.0 ± 1 °C and 0.2 U/L yogurt starter culture was added. For yogurt production, milk was transferred to 400-g glass yogurt containers and incubated for approximately 4 h until the pH reached 4.6. The yogurt, which reached the target pH level, was quickly cooled and kept in the refrigerator at 6–8 °C for one day before being analyzed.

#### 2.2.3 Cheese production

For the cheese production heat-treated goat milk was cooled to 38.0 ± 1 °C, then 0.02% CaCl₂ and 0.1 U/L cheese starter culture was added into the milk. Milk was coagulated by adding 1 mL of animal rennet per 10 L of milk. Curd was cut into approximately 3 cm^3^ cubes using curd-cutting tools after 75 min clotting. Curd was transferred into cheesecloth and a whey release step was carried out (self-draining for 20 min and pressing (approximately 3100 Pa for the first 30 min and about 6200 Pa for the following 60 min) for 90 min). The cheese obtained was kept in a brine with a salt content of 12% at 27.5 ± 1 °C for 12–14 h, then removed from the brine to dry the surfaces and dried with a fan for approximately 1 h. The dried cheeses were packaged in plastic vacuum bags and kept in the refrigerator at 6–8 °C for one day before being analyzed. The flow chart for production is presented in Fig. 1.

### 2.3 Determination of physicochemical characteristics and nitrogen fractions

#### 2.3.1 Determination of physicochemical characteristics

DM was determined by the standard drying method at 102 ± 2 °C (AOAC International, 2000; NEN, 1969), fat content was measured using the Van Gulik method (AOAC International, 2000; NEN, 1969) and total nitrogen (TN) was analyzed by the micro-Kjeldahl method (AOAC International, 2000; NEN, 1969). Protein content was calculated as TN multiplied by 6.38. Titratable acidity (TA) as% of lactic acid was determined. pH measurements were carried out using pH meter (Sartorius, PB-11, Göttingen, Germany).

#### 2.3.2 Determination of water-soluble nitrogen (WSN)

The WSN fractions were prepared according to the method suggested by Kuchroo and Fox (1982). For this purpose, kefir, yogurt and 1:2 cheese water mixture at 40 °C were homogenized for 2 min with the help of a blender (IKA-WERK, Staufen, Germany) and then kept in a water bath (40 °C, 1 h). Prepared samples were centrifuged at +4 °C at 3000*g* for 30 min (Eppendorf 5810 R, Hamburg, Germany), the upper fat layer was removed, and the liquid part was filtered through Whatman No: 42 filter paper. Extracts were stored at −20 °C, until analysis. The nitrogen content of WSN fraction, expressed as percentage of TN, were determined by micro-Kjeldahl method (AOAC International, 2000).

#### 2.3.3 Determination of 12% trichloroacetic acid-soluble nitrogen (TCA-SN)

The TCA-SN of samples was determined in the WSN fraction of the samples. About 25 mL of the WSN fraction was taken, and an equal volume of 24% trichloroacetic acid (TCA) solution (resulting in a final TCA concentration of 12%) was added. After waiting at room temperature for 2 h, 25 mL of the filtrate was filtered through Whatman No: 42 filter paper and the nitrogen content dissolved in TCA, expressed as percentage of TN, was determined by the micro-Kjeldahl method (AOAC International, 2000; Polychroniadou, Michaelidou, & Paschaloudis, 1999).

#### 2.3.4 Determination of total free amino acid (TFAA)

The TFAA of WSN fraction of samples were determined according to the spectrophotometric method specified (Folkertsma & Fox, 1992). For this purpose, 10–100 mL of sample (according to the expected amount of free amino acids) was mixed with 1 mL of water and then 2 mL of Cadmium-ninhydrin (0.8 g of ninhydrin was dissolved in 80 mL of ethanol and 10 mL of acetic acid and prepared by adding 1 mL of CdCl_2_ dissolved in water to the resulting mixture) was added to the mixture. The resulting mixture was kept at 84 °C for 5 min and then cooled, and the measurement was made on a UV-spectrophotometer (UV-1800 UV–vis, Shimadzu, Tokyo, Japan) at a wavelength of 507 nm. The absorbance values obtained were calculated as mg leucine (leu)/g of sample using the standard curve to be prepared with the leu.

### 2.4 Simulated *in vitro* digestion

*In vitro* digestion of cheese samples was carried out according to a standardized static *in vitro* digestion method suitable for food developed by Minekus et al. (2014) using the enzymes pepsin from porcine gastric mucosa (≥25,000 U/mL), pancreatin from porcine pancreas 8xUSP (≥800 U/mL) and porcine bile extract (160 mM). The oral phase of the *in vitro* digestion process was omitted because the samples were fermented products and the research primarily targeted protein digestion. For the analysis 5 g of sample (2.5 g sample + 2.5 g water for cheese sample) was incubated with 5 mL of simulated salivary fluid at 37 °C for 2 min by shaking. Simulated gastric fluid was added to the mixture obtained at the end of incubation and the pH was adjusted to 2.5–3 with 1 M HCl. The digestion mixture was incubated in a rotary shaker at 37 °C for 2 h by adding 1.28 mL of pepsin. After incubation, the pH of the samples was adjusted to 7.5 using 1 M NaOH and then intestinal fluid (7.7 mL intestinal fluid +3.5 mL pancreatin solution +1.75 mL bile solution) was added. The solution was incubated for 2 h in a rotary shaker at 37 °C. At the end of digestion, the process was terminated by placing the samples in ice water and then diluted to pH 7 with cold 0.1 mol/L potassium phosphate buffer at a ratio of 1:5. Pure water was used instead of the sample as a blank. A 2 mL digestion sample was taken after the oral and gastric phases. After the *in vitro* digestion procedure, a centrifuge process was carried out at 4 °C and 9000 rpm for 30 min, and the middle layers of centrifuged samples (post pancreatic fraction) were used for the determination of the degree of hydrolysis (DH), antioxidant activity analyses and isolation of low molecular weight (<3 kDa) fractions.

### 2.5 Isolation of low molecular weight (<3 kDa) fractions

The LMP containing potential bioactive peptides of the day-1 samples was obtained by ultrafiltration because these samples exhibited the highest overall antioxidant activity based on the antioxidant activities determined in the post-pancreatic fractions of all storage-day samples. Fractionation was carried out at 4 °C and 4600 rpm using Vivaspin Turbo 15 (Sartorius Stedim Biotech, Göttingen, Germany) centrifugal filter units with a cut-off of 3 kDa (3000 MWCO). The low molecular weight (<3 kDa) fractions were used for the determination of antioxidant activity analyses.

### 2.6 Determination of degree of hydrolysis (DH)

The DH of goat milk, kefir, yogurt and cheese samples was determined using the OPA (*o*-phthaldialdehyde) method as a result of *in vitro* gastrointestinal digestion. For the analysis, the OPA reagent was first prepared: 7.62 g of sodium tetraborate decahydrate and 200 mg of sodium dodecyl sulfate were dissolved in 150 mL of distilled water. About 160 mg of OPA (dissolved in 4 mL of ethanol) and 176 mg of dithiothreitol were added to make the volume up to 200 mL. Serine (0.1–1 mg/mL range) amino acid was used as a standard. In the analysis, 400 μL of serine standard, distilled water (blank), or sample were added to tubes containing 3 mL of OPA reagent, respectively, and the tubes were left to stand at room temperature for 2 min after mixing. Absorbance was measured at 340 nm on a spectrophotometer (UV-1800 UV–vis, Shimadzu, Tokyo, Japan). The results were converted into the amount of free amino groups using the standard curve, and the DH were calculated according to the method (Nielsen, Petersen, & Dambmann, 2001). DH data were corrected with the data obtained in the substrate free control digestion.

### 2.7 Determination of antioxidant activities

The antioxidant activities were assessed in post-pancreatic fractions from samples stored for 1, 14, and 28 days, and also in low-molecular-weight peptide (LMP) fractions, using FRAP (Ferric Reducing Antioxidant Power) and CUPRAC (Cupric Ion Reducing Antioxidant Capacity) methods (Apak, Güçlü, Özyürek, & Karademir, 2004; Benzie & Strain, 1996).

#### 2.7.1 FRAP method

For FRAP analysis, a buffer solution was prepared by mixing 0.1 mol/L acetate (pH 3.6), 10 mmol/L 2,4,6-tris(2-pyridyl)-*s*-triazine and 20 mmol/L FeCl₃·6H₂O solutions at 10:1:1 ratio. About 50 μL of sample was taken, 50 μL of buffer was added and mixed, and the mixture was kept in the dark for 30 min. Then, absorbances were measured at a wavelength of 593 nm (Tecan Infinite M200, Männedorf, Switzerland) Trolox standard was analyzed at different concentrations and linear regression analysis was applied by plotting the absorbance values obtained against sample amounts. Standard curve and the equation defining this curve were determined. The results were calculated using the Trolox standard curve and expressed as mM Trolox/g protein.

#### 2.7.2 CUPRAC method

In order to determine antioxidant activity according to the copper(II) ion reduction based antioxidant capacity (CUPRAC) measurement method in samples, 10 mM copper(II) solution CuCl₂·2H₂O, 7.5 mM neocuproine solution and 1 M ammonium acetate solution were prepared. About 70 μL copper (II) solution (10 mM), 70 μL neocuproine solution (7.5 mM) and 70 μL ammonium acetate buffer (1 M) were added into the microplate wells, respectively, and 7 μL sample was added, followed by 70 μL distilled water. The microplate was shaken well for 10 s, sealed and kept at room temperature for 30 min. At the end of 30 min, absorbance was measured at a wavelength of 450 nm (Infinite M200) against the blank. The results were calculated using the Trolox standard curve and expressed as mM Trolox/g protein.

### 2.8 Statistical analysis

Data were obtained by performing two replicate productions and at least two parallel analyses for each parameter. Kefir, yogurt, and cheese or samples on the same storage day or samples during storage were compared using One-way ANOVA. Comparison of means was carried out by using Tukey's or Welch's (for the non-parametric data) test. Data were evaluated by SPSS 25.0 for windows (SPSS, Chicago, IL, USA) software. Results were expressed as mean ± standard error to indicate the precision of the sample mean and account for variability in the data.

## 3 Results and discussion

### 3.1 Chemical composition of kefir, yogurt, and cheese

The dry matter, fat and protein contents of kefir, yogurt, and cheese stored for first day are presented in Table 1. Fat and protein results of samples are also presented as fat in DM (fat/DM) and protein in DM (protein/DM) (Table 1). The kefir, yogurt, and cheese samples were produced at a commercial dairy plant using routine manufacturing process for commercial products. Thus, the standardization applied to the milk used for yogurt and kefir production. Cheese production, characterized by whey separation, resulted in high DM, fat, fat/DM, protein and protein/DM, while the standardization applied to the milk used for yogurt and kefir production directly influenced the compositional characteristics of these products.

**Table 1:** Chemical composition of kefir, yogurt and cheese.

Parameter	Kefir	Yogurt	Cheese
DM%	13.89 ± 0.04	17.61 ± 0.05	40.33 ± 0.15
Fat%	4.60 ± 0.10	6.40 ± 0.05	18.75 ± 0.25
Fat/DM%	33.13 ± 1.17	36.34 ± 0.28	46.49 ± 0.45
Protein%	4.77 ± 0.08	6.75 ± 0.17	18.77 ± 0.14
Protein/DM%	34.32 ± 0.63	38.33 ± 1.26	46.54 ± 0.17

Values are mean ± standard error of the samples. DM, dry matter; Fat/DM, fat in dry matter; Protein/DM, protein in dry matter.

According to the results, the DM contents were determined as 13.89% for kefir, 17.61% for yogurt, and 40.33% for cheese. The highest DM content was observed in the cheese sample while the lowest was found in kefir sample as expected. A recent study reported that goat kefir contained 14.58 g/100 g DM (Muñoz-Bas et al., 2025). The difference between our finding and those Muñoz-Bas et al. may be attributed to difference in standardization of DM. Additionally, the yogurt sample contained 17.61% DM. Similarly, according to a study, goat yogurt had a DM content of 14.52% (Ibrahim, Naufalin, Muryatmo, & Dwiyanti, 2021). In addition, a recent study indicated that goat cheese contained 46.10% DM (Pajor et al., 2023). The DM content of cheese obtained in this study is consistent with the range reported in the literature and is slightly lower than the value reported in our previous study (Uzkuç & Karagül Yüceer, 2023). In the referenced study (Uzkuç & Karagül Yüceer, 2023), the DM content of goat cheese ranged from 38.56% to 48.12%, while in the current study it was measured as 40.33%. The changes in DM among cheeses could be attributed to variations in milk composition, particularly fat and protein content, or differences in moisture retention during cheese storage. In comparison, a recent study reported that the DM content ranged from 42.02% to 45.14%, which aligns with our findings and supports the typical variability observed across different production processes (Kawęcka & Pasternak, 2024). However, another study reported that the DM content of goat cheese ranged from 38.91% to 52.64% during the ripening process (day 0 to day 28) (Mladenović et al., 2021).

The goat milk kefir contained 4.60% fat, which is consistent with the findings of a recent study which reported 4.87% fat content in goat kefir (Muñoz-Bas et al., 2025). This is also in agreement with the study by Wang et al. (2023), who reported a fat content of 4.15 g/100 g. Wanniatie, Qisthon, Husni, and Yuliawan (2022) reported a similar fat content in goat yogurt (6.57%) to that observed in our study (6.40%), whereas Gursel et al. (2016) reported a lower value (3.90%). The differences in gross  composition of goat kefir and yogurts may be attributed to production-related factors such as the type of milk used, fermentation cultures, and processing methods. According to a study, the goat cheeses produced using different starter cultures had a fat content ranging from 14.3 to 22.5% and a fat/DM ratio between 49.8 and 52.4% (Pappa, Kondyli, Bosnea, Malamou, & Vlachou, 2022). Similarly, in our study, the cheese sample contained 18.75% fat and fat/DM ratio of 46.49%. Our results are partially consistent with those of Kawęcka and Pasternak (2024), who reported that fat content varied between 17.51% and 20.58%, depending on the goat breed. In the study conducted by Pajor et al. (2023), a fat content of 22.14% and a fat/DM ratio of 48.37% were reported. A slight difference was observed compared to our study, it may because of milk content.

A study found that the protein content of goat kefir is 4.11–4.92%, which is consistent with our result of 4.77% (Wulansari, Widodo, Sunarti, & Nurliyani, 2023). Regarding the protein content of goat yogurt, research revealed that 3.18% and 3.51% (Ibrahim et al., 2021; Wang et al., 2023). In contrast, the goat yogurt produced in our study had a significantly higher protein content 6.75%. This discrepancy could be attributed to the type of yogurt and factors including milk composition. In addition, milk standardization (evaporation processes), as well as seasonal factors, including milk collection during late lactation can directly contribute to the product composition. Milk collected at the end of lactation (late October in our case) typically contains higher fat and protein levels due to physiological changes in the mammary gland. This difference in composition, in addition to breed and feeding practices, may contribute to the observed differences in fat and protein content. In the case of goat cheese, previous reports indicate a wide protein content range from 9.8% and 20.35% depending on ripening and storage time (Kawęcka & Pasternak, 2024; Pajor et al., 2023; Pappa et al., 2022). The protein content of goat cheese in our study was 18.77%, which falls within the range reported by Kawęcka and Pasternak (2024). This alignment indicates a typical protein concentration for fresh to mid-ripened goat cheeses and may reflect comparable production conditions and milk quality. Additionally, the protein/DM ratio was previously reported to range from 45.39 to 49.33, whereas in this study it was found to be 46.54 (Uzkuç & Karagül Yüceer, 2023). As reported in previous studies, factors such as the composition of the milk, the type and amount of starter culture, including its specific bacterial composition, and the applied processing method, are considered potential contributors to the differences in the chemical composition of the final products.

### 3.2 Physicochemical properties of kefir, yogurt, and cheeses during storage

**Fig. 2:**
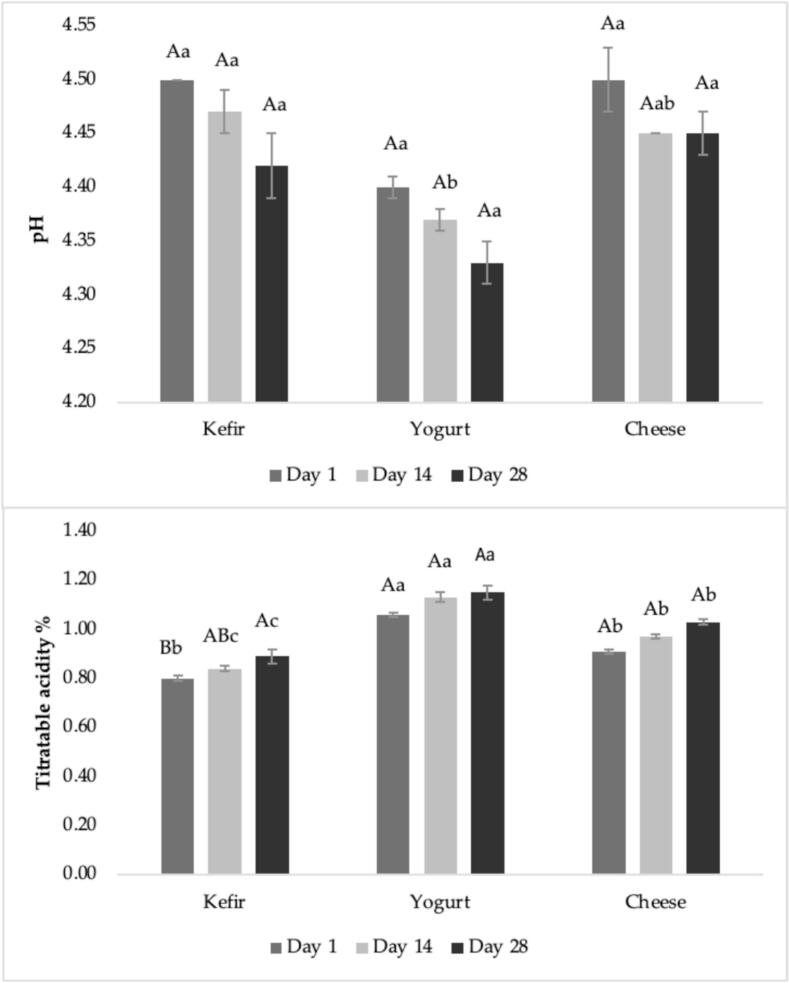
pH and titratable acidity of kefir, yogurt and cheese during storage at 6–8 °C. Statistically significant (*P* ≤ 0.05) differences among the mean values were indicated by different lowercase letters (for differences among the samples on the same storage day) and different uppercase letters (for changes in each sample during storage).

The pH, and TA of kefir, yogurt and cheese on days 1, 14 and 28 of storage are presented in Fig. 2. In kefir samples, a decrease in pH (from 4.50 to 4.42) was observed during storage indicating an increase in acidity (from 0.80 to 0.89), however the changes were not significant (*P* > 0.05). Similar, a recent study reported a pH value of 4.32 for goat milk kefir (Muñoz-Bas et al., 2025). Similar to our findings, another study also noted that pH decreased and acidity increased with extended incubation, reporting pH values ranging from pH 4.16 to 5.40 (Sulmiyati, Said, Fahrodi, Malaka, & Fatma, 2019). The pH of goat yogurt was observed to decrease from 4.40 to 4.33 over the storage period (days 1, 14 and 28), however this decrease was not significant (*P* > 0.05). Similarly, research reported pH value between 4.34 and 4.49 for goat yogurt (Ibrahim et al., 2021; Jrad, Oussaief, El-Hatmi, & Bouaziz, 2022; Wang et al., 2023). Although the observed decrease in pH during storage was not significant, this slight decline may still be attributed to post-acidification. In the present study, similar to other products, the pH value of goat cheese was found to decrease from the 1st to the 14th day of storage (from 4.50 to 4.45), remain constant between the 14th and 28th days (4.45 to 4.45), and such a decreasing trend is consistent with that observed during cheese ripening (Uzkuç & Karagül Yüceer, 2023). Consistent results were also presented in a study, pH values of 4.79 and 4.86 for fresh goat cheese on day 1, with no significant changes during storage (Pappa et al., 2022). The decrease in pH values has been associated with the acidification as a result of bacterial activity during cheese ripening.

As indicated in Fig. 2, TA values increased in all products (kefir, yogurt, and cheese) throughout the storage period (days 1, 14 and 28), however the changes in kefir were significant (*P* ≤ 0.05). Among the three, yogurt exhibited the highest TA values, followed by cheese and kefir, respectively. This increase is primarily attributed to production during lactose fermentation by lactic acid bacteria. A recent study revealed that a TA value of 94.00 Dornic (°D) for kefir, corresponding to approximately 0.85%, which aligns closely with our kefir values: 0.80% (day 1), 0.84% (day 14), and 0.89% (day 28) (Muñoz-Bas et al., 2025). Similarly, another study documented TA values between 0.73% and 0.79% for goat kefir during storage, consistent with our findings (Kef & Arslan, 2021). It was found that TA values increased progressively during storage (Yang, Zhang, Zhang, Wang, & Liu, 2023). In our study, goat yogurt TA was 1.06% (day 1), 1.13% (day 14), and 1.15% (day 28), which shows a minor difference from Yang et al.'s values. For goat cheese, TA values in our study ranged between 0.91% and 1.03% during the storage period. In our previous study, this value was recorded as 1.04 (Uzkuç & Karagül Yüceer, 2023). Another research reported TA values for goat cheese ranging from 0.08% to 1.20%, demonstrating broader variation across production conditions (Say, 2022).

TN value evaluates the products in terms of protein and thus reveals their nutritional value. In our study, no statistically significant difference was found in TN values among the samples during storage. In the meantime, the TN content of goat kefir was observed to increase during storage, from 0.75% on day 1 to 0.86% on both days of 14 and 28. On the other hand, yogurt samples exhibited a slightly smaller increase in TN compared to kefir samples, with values rising from 1.06% on day 1 to 1.07% on both days 14 and 28. In our previous study, the TN content of goat cheese ranged from 2.6% to 3.6% (Uzkuç & Karagül Yüceer, 2023). These values are consistent with those obtained in the current study, which ranged from 2.94% to 2.99%. Similarly, a study reported comparable TN values, between 2.61 g/100 g and 3.09 g/100 g (Zaravela, Kontakos, Badeka, & Kontominas, 2021). Although different starter cultures were used in the goat cheeses produced in a recent study, no significant differences in TN content were observed (ranging from 1.5 to 2.3%) (Pappa et al., 2022). However, a slight difference was identified when compared to our study. The results of both studies suggest that despite the initiation of proteolysis, no significant reduction in TN content occurred.

As a biochemical indicator of proteolysis, WSN levels during storage were evaluated in all products examined in our study (Table 2). To better assess the extent of proteolysis, the WSN/TN (%) ratio was also evaluated. This allowed for more accurate comparisons between samples, including those produced with different cultures or stored for different durations. Both WSN and WSN/TN values were significantly higher in cheese samples compared to kefir and yogurt (*P* ≤ 0.05). In kefir samples, WSN values were 0.09 on day 1, 0.11 on day 14, 0.12 on day 28, with no statistically significant differences observed across the storage period. WSN/TN values ranged from 12.56% to 13.49%. In yogurt samples, WSN values were similarly stable: 0.10 on day 1, 0.12 on day 14, and 0.13 on day 28. WSN/TN values in yogurt ranged from 9.82% to 12.01%. Kefir and yogurt samples did not show any statistically significant changes in WSN/TN values during storage (*P* > 0.05), and no significant differences were observed between them. A study revealed that WSN values were between 0.133% and 0.153% (Niamah, 2017). In contrast, in cheese samples, the WSN content was observed to increase progressively during the 28-day storage period. Similarly, the WSN/TN (%) ratio showed a consistent rise, from 16.86 on day 1, 18.99 on day 14, and 20.41 on day 28, indicating ongoing proteolytic activity and accumulation of soluble nitrogen compounds. In a comparable study, it was found that WSN/TN values ranging from 12.95% to 19.41%, which support the notion that proteolytic activity develops over time during cheese ripening (Pappa et al., 2022). Another research found that as the fat content in goat cheese decreased, WSN/TN (%) ratio also decreased. For instance, cheeses containing 1.4%, 2.5%, and 4.3% fat had corresponding WSN/TN values of 6.94%, 8.87%, and 10.54% respectively (Zaravela et al., 2021). Additionally, the inclusion of adjunct cultures such as *Lactobacillus helveticus* was reported to improve proteolysis and increase WSN/TN ratio. These findings provide further evidence that the starter cultures can significantly influence the extent of proteolysis during ripening.

**Table 2:** Nitrogen fractions of kefir, yogurt and cheese during storage at 6–8 °C.

Parameter	Day	Kefir	Yogurt	Cheese
TN%	1	0.75 ± 0.01^Ac^	1.06 ± 0.03^Ab^	2.94 ± 0.02^Aa^
	14	0.86 ± 0.03^Ac^	1.07 ± 0.03^Ab^	2.99 ± 0.02^Ac^
	28	0.86 ± 0.03^Ac^	1.07 ± 0.03^Ab^	2.98 ± 0.02^Aa^
WSN%	1	0.09 ± 0.01^Ab^	0.10 ± 0.01^Ab^	0.50 ± 0.00^Ca^
	14	0.11 ± 0.01^Ab^	0.12 ± 0.01^Ab^	0.57 ± 0.00^Ba^
	28	0.12 ± 0.01^Ab^	0.13 ± 0.01^Ab^	0.61 ± 0.00^Aa^
WSN/TN%	1	12.59 ± 0.87^Ab^	9.82 ± 0.43^Ab^	16.86 ± 0.24^Ba^
	14	12.56 ± 0.64^Ab^	11.10 ± 0.40^Ab^	18.99 ± 0.29^Aa^
	28	13.49 ± 0.69^Ab^	12.01 ± 0.50^Ab^	20.41 ± 0.25^Aa^
TCA-SN/TN%	1	10.99 ± 0.14^Aa^	7.80 ± 0.22^Bb^	7.13 ± 0.10^Cb^
	14	11.14 ± 0.34^Aa^	9.01 ± 0.26^ABb^	8.17 ± 0.12^Bb^
	28	12.64 ± 0.39^Aa^	10.25 ± 0.31^Ab^	9.26 ± 0.15^Ab^
TFAA (mg leu/g sample)	1	0.50 ± 0.03^Cb^	0.35 ± 0.00^Cc^	1.54 ± 0.02^Ca^
	14	1.12 ± 0.07^Bb^	0.79 ± 0.00^Bc^	3.44 ± 0.05^Ba^
	28	2.39 ± 0.15^Ab^	1.70 ± 0.01^Ac^	7.37 ± 0.10^Aa^

Values are mean ± standard error of the samples. TN, total nitrogen; WSN, water-soluble nitrogen; TCA-SN, trichloroacetic acid-soluble nitrogen; TFAA, total free amino acids. Statistically significant (*P* ≤ 0.05) differences among the means were marked with different lowercase letters in each row and different uppercase letters during storage for each parameter.

TCA-SN/TN was utilized to evaluate nitrogenous compounds, including small peptides and free amino acids, generating through protein degradation. Among the three products, the TCA-SN/TN ratio was highest in kefir, followed by yogurt and cheese. In kefir, the ratio increased from 10.99% on day 1 to 12.64% on day 28. For yogurt, values were 7.80% on day 1 and 10.25 on day 28. In our previous studies, TCA-SN/TN ratio in goat cheese samples ranged from 7.13% to 9.26%. In a study, a positive correlation was identified between the TCA-SN percentage and fat content of goat cheese, with values reported between 5.15% and 7.76% (Zaravela et al., 2021). Specifically, the TCA-SN/TN value was 6.74% when adjunct culture was added and 6.01% when was not added. This increase over time was attributed to proteolysis during ripening. Similarly, another study reported TCA-SN/TN values in goat cheese ranging from 6.59% to 12.80% throughout storage (Pappa et al., 2022). These findings are consistent with the results of our studies.

The ripening process was evaluated by measuring the TFAA released as a result of proteolysis. As shown in Table 2, the TFAA content of kefir samples ranged from 0.50 mg leu/g of kefir to 2.39 mg leu/g of kefir on days 1 and 28. According to a study, the TFAA content of kefir samples was reported as L-leucine equivalent in the range of 0.08–0.17% (*w*/*v*) (İnce-Coşkun & Özdestan-Ocak, 2022). Furthermore, they noted a positive correlation between TFAA levels and the formation and accumulation of biogenic amines. These findings suggest that TFAA is not only an indicator of proteolysis but also serves as a quality and safety parameter in fermented dairy products. Among all samples, yogurt exhibited the lowest TFAA: 0.35 mg leu/g of yogurt on day 1 of storage, 0.79 mg leu/g of yogurt on day 14, and 1.70 mg leu/g of yogurt on day 28. In a study, the TFAA content of goat milk fermented with different bacterial strains and fermentation times was investigated (Akan, 2022). Among the single-strain applications, *Lactobacillus acidophilus* produced the highest TFAA level, reaching of 182.98 μg/mL at leu after 12 h of fermentation. *Lactobacillus casei* and *Bifidobacterium longum* reached 137.66 μg/mL leu and 64.05 μg/mL leu, respectively, both after 6 h of fermentation. When mixed cultures were used, *L. casei* combined with *S. thermophilus*, yielded 123.16 μg/mL leu was reported after 8 h, while *L*. *acidophilus* and *S. thermophilus* reached 143.81 μg/mL leu, and *B. longum* and *S. thermophilus* reached 109.08 μg/mL leu at the same time point. These findings demonstrate that TFAA levels were significantly influenced by both the bacterial species and duration of fermentation. In our cheese samples, the TFAA content increased progressively during storage, reaching 1.54 mg leu/g of cheese on day 1, 3.44 mg leu/g of cheese on day 14 and 7.37 mg leu/g of cheese on day 28. These findings are consistent with those reported in our previous study, where the TFAA values ranged from 0.767 mg leu/g of cheese to 8.800 mg leu/g of cheese, which are similar to our study (Uzkuç & Karagül Yüceer, 2023). However, another study revealed that the TFAA content of Murcia al Vino goat cheese was significantly influenced by the type of coagulant used (Abellán et al., 2012). Their results, which ranged from 735 to 854 mg/100 g DM, corresponding approximately to 3.0–3.5 mg leu/g of cheese and showed slightly lower TFAA levels compared to our findings.

### 3.3 Degree of hydrolysis

**Fig. 3:**
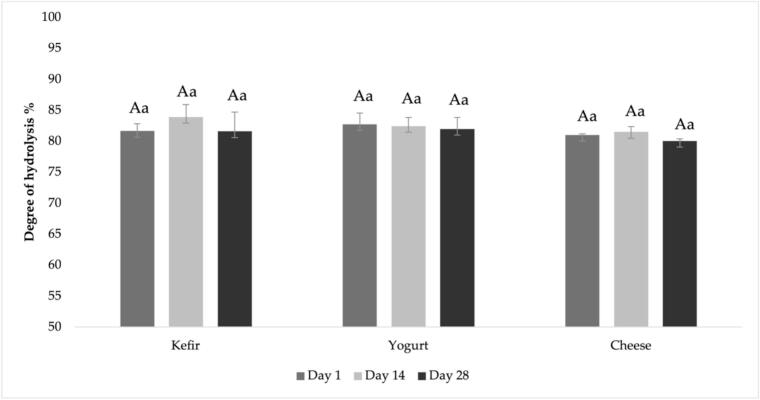
Hydrolysis degree of kefir, yogurt and cheese during storage 6–8 °C. Statistically significant (*P* ≤ 0.05) differences among the mean values were indicated by different lowercase letters for differences among the samples on the same storage day, and by different uppercase letters for changes in each sample during the storage.

Proteolysis is the hydrolysis of large and complex proteins into smaller and simpler peptides. Enzymatic hydrolysis improves the functional properties of dietary proteins by converting them into bioactive peptides while preserving their nutritional value (Uzkuç & Yüceer, 2025). The digestion process has the noteworthy potential that proteolytic digestive enzymes can release bioactive peptides from goat milk proteins (Tagliazucchi, Helal, Verzelloni, Bellesia, & Conte, 2016). The hydrolysis of the goat kefir, yogurt, and cheese protein and peptides after the *in vitro* digestion was evaluated by consulting the OPA assay based on the measurement of free NH_2_ groups (serine equivalent). Fig. 3 shows the resulting DH, total of proteolysis in the samples before digestion and protein hydrolysis after *in vitro* digestion. Based on the results of WSN/TN, TCA-SN/TN, and TFAA, although significant differences were observed in proteolysis levels prior to *in vitro* digestion (Table 2), the total DH of goat milk proteins after *in vitro* digestion was not significantly affected by the production of kefir, yogurt, or cheese (*P* > 0.05). After 2 h of peptic and 2 h of pancreatic digestion, the average DH of kefir, yogurt and cheeses were 82.37, 82.39, and 80.84%, respectively. In a study, the OPA method was used to determine DH, and kefir samples showed 83.6% DH at the end of digestion, similar to kefirs in our study (Nehir El et al., 2015).

The DH fluctuated during storage and no general trend was found for the release of free NH_2_ groups by *in vitro* digestion in samples on different storage days. However, DH did not change significantly during storage (*P* > 0.05). In a study examining the amount of peptide released from Ras cheese through *in vitro* digestion, it was reported that, similar to our study, there was no significant difference between cheeses ripened for different periods (Helal, Cattivelli, Conte, & Tagliazucchi, 2023).

During *in vitro* digestion, digestive enzymes such as pepsin and pancreatin play a crucial role in breaking down milk proteins into peptides and amino acids. Pepsin initiates protein hydrolysis in the gastric phase, while pancreatin further degrades proteins in the intestinal phase, resulting in smaller peptides and increased free amino acids, which are important for absorption and bioactivity. The structure of the milk protein matrix also affects digestion; for example, casein micelles form curds that slow hydrolysis, while whey proteins are digested more rapidly (Halabi, Croguennec, Bouhallab, Dupont, & Deglaire, 2020; Mulet-Cabero et al., 2020). Therefore, relatively low DH values in cheese samples can be attributed to higher casein derived peptides as a result of whey discharge during cheesemaking.

Proteases (endogenous milk enzyme, enzyme of starter microorganism, and rennet enzymes for rennet cheese) may have remained active after processing and may have contributed to protein hydrolysis in addition to the added digestive enzymes. The presence of hydrolyzed proteins in products can enhance initial digestion rates without affecting protein coagulation (Lambers, Wissing, & Roggekamp, 2023). However, compared to digestive enzymes like pepsin and pancreatin, the effect of microorganisms and rennet on protein breakdown is smaller; digestive enzymes are responsible for the majority of protein hydrolysis, while microorganisms and rennet mainly influence the types of peptides produced and the bioactivity of the digested products (Dos Santos Aguilar & Sato, 2018; Huppertz & Chia, 2021). In accordance, DH values were found to be similar after *in vitro* digestion of all samples, even though they were produced using different starters and production methods.

### 3.4 Antioxidant activities of kefir, yogurt, and cheeses

The antioxidant activities of digested samples obtained through CUPRAC and FRAP assays are presented in Table 3. Similar DH were achieved in kefir, yogurt and cheese samples subjected to *in vitro* digestion (Fig. 3).

**Table 3:** Antioxidant activities of digested (post-pancreatic) kefir, yogurt and cheese.

Method	Day	Kefir	Yogurt	Cheese
CUPRAC (mM Trolox/g protein)	1	116.05 ± 10.37^Aa^	123.30 ± 5.58^Aa^	89.35 ± 6.29^Aa^
	14	107.94 ± 2.45^Aa^	93.68 ± 3.02^Aa^	87.54 ± 2.70^Aa^
	28	100.29 ± 5.18^Aa^	96.10 ± 7.04^Aa^	79.40 ± 3.15^Aa^
FRAP (mM Trolox/g protein)	1	14.22 ± 0.43^Ab^	11.40 ± 0.23^Ab^	22.74 ± 0.09^Aa^
	14	12.68 ± 0.37^Ab^	14.12 ± 0.73^Ab^	18.96 ± 0.75^Ba^
	28	16.61 ± 0.31^Aa^	12.24 ± 0.14^Ab^	18.03 ± 0.29^Ba^

Values are mean ± standard error of the samples. Statistically significant (*P* ≤ 0.05) differences among the means were marked with different lowercase letters in each row and different uppercase letters during storage for each parameter.

Regarding digested samples, the highest antioxidant capacity based on the CUPRAC method was observed in the yogurt samples on the first day of storage, although the difference was not statistically significant. However, FRAP results were not found to be consistent with this finding. The inconsistent trends observed between CUPRAC and FRAP experimental results can be attributed to fundamental differences in method principles, pH conditions, and sensitivities to different classes of antioxidants (*e.g.*, peptides, thiol groups) present in the yogurt matrix (Karadag, Ozcelik, & Saner, 2009).

It has been shown that antioxidant peptides can be released from caseins during hydrolysis by digestive enzymes or milk fermentation by proteolytic LAB strains. Not every peptide exhibits bioactive properties; these properties may vary depending on the peptide's amino acid sequence, structural stability, and interaction with cellular targets (Korhonen & Pihlanto, 2006).

According to CUPRAC results, an insignificant decrease in the antioxidant activity of kefir was determined during storage (*P* > 0.05), with values of 116.05 mM Trolox/g protein on day 1, 107 mM Trolox/g protein on day 14 and 100.29 mM Trolox/g protein on day 28 (Table 3). The difference between the groups is not statistically significant. In contrast, FRAP values exhibited a non-linear trend, with values of 14.22, 12.68, and 16.61 mM Trolox/g protein on days 1, 14, and 28, respectively.

The mesophilic/thermophilic kefir culture used in this study, which contains various bacterial and yeast species, may have contributed to diverse biological activities during storage. The observed decrease in antioxidant capacity in the CUPRAC assay may be related to degradation or to structural changes in peptides over time. The decrease in antioxidant capacity observed in digested samples during storage may be attributed to the comparatively lower antioxidant potential of peptides released through proteolysis. The initially high antioxidant capacity could be associated with the presence of lactic acid bacteria found in the products (Li, Liu, & He, 2015). Beyond bioactive peptides, the antioxidant potential of fermented dairy products can also derive from viable microorganisms themselves, which are capable of scavenging free radicals or producing antioxidant metabolites (Gao et al., 2025). Additionally, yeast species such as *D. hansenii* may have contributed to the results by enhancing both peptide production and the synthesis of redox-active metabolites during fermentation. Variations in FRAP analysis during the storage period may reflect dynamic changes in the concentration and composition of antioxidant compounds, including amino acids, small peptides, and organic acids.

Various studies have shown that *in vitro* digestion significantly increases the antioxidant activity of kefir, largely as a result of the bioactive peptides release. For instance, a study reported an increase from 437 μM ascorbic acid equivalent/mg protein to 2867 μM ascorbic acid equivalent/mg protein using the phosphomolybdenum reduction assay (Nehir El et al., 2015). Similarly, another study observed an increase in DPPH radical scavenging activity from 4.2% to 63.06%, while a decrease in FRAP values was reported, likely due to the method's sensitivity to specific redox mechanisms (Üstün-Aytekin, Şeker, & Arısoy, 2020). A recent study also reported a progressive increase in antioxidant activity measured by the CUPRAC assay following digestion (Saygili & Karagozlu, 2025). While these investigations demonstrate the positive influence of digestion, our findings indicate that the antioxidant activity of digested kefir may gradually decrease during storage.

Yogurt samples maintained a moderate but consistent antioxidant profile over the storage period (Table 3). Based on CUPRAC assay findings, the highest antioxidant activity in digested yogurt samples was recorded on the first day of storage, measured as 123.30 mM Trolox/g protein. Additionally, the highest FRAP value was detected on the 14th day, reaching 14.12 mM Trolox/g protein. Despite these fluctuations, statistical analysis revealed no significant differences among storage days (*P* > 0.05). The variation in antioxidant activity observed during storage days may be attributed to the proteolytic potential of the thermophilic yogurt culture used (Settachaimongkon et al., 2014). The initially high CUPRAC value on day one may be due to the early formation of bioactive peptides with antioxidant properties during fermentation by thermophilic starter cultures (*L. delbrueckii* ssp. *bulgaricus* and *S. thermophilus*).

These peptides are known to possess functional properties such as free radical scavenging and metal binding (chelating), which contribute to the determined antioxidant capacity. Regarding CUPRAC assay, the decrease on day 14 may be related to the degradation of peptides or their conversion to less potent compounds as a result of ongoing enzymatic and proteolytic processes during storage (Uzkuç & Yüceer, 2025). Another notable finding is the slight increase in antioxidant activity observed on day 28. This may indicate the formation or accumulation of smaller peptides or low-molecular-weight compounds with antioxidant activity. Since these changes were not statistically significant, the results should be interpreted with caution.

The strong proteolytic activity in cheese, driven by *Lc. lactis* ssp. *lactis* and *Lc. lactis* ssp. *cremoris* cultures along with rennet-derived proteases (chymosin+pepsin), led to the release of a wide range of peptides, which initially resulted in high antioxidant activity as measured by both CUPRAC and FRAP assays. However, during storage, the *in vitro* digested antioxidant activity exhibits a decreasing trend, likely due to further breakdown or inactivation of peptides. This decline, particularly in reducing capacity, may be attributed to the structural degradation of peptides. In support of this, TFAA content increased from 1.54 to 7.37 (mg leu/g sample) over the storage period, indicating a significant accumulation of peptides and free amino acids (Table 2). Similarly, the increase in WSN/TN and TCA-SN/TN ratios pointed to the progressive formation of peptides and low-molecular-weight protein fragments. The CUPRAC value of cheese, which was 89.35 mM Trolox/g protein at the beginning of storage, decreased to 79.40 mM Trolox/g protein by day 28; however, this reduction was not statistically significant. Since CUPRAC primarily detects the reducing capacity of peptides containing thiol groups or phenolic amino acids, such stability of CUPRAC values suggests partial retention of reducing groups. On the other hand, FRAP values showed a more noticeable decline, from 22.74 to 18.03 mM Trolox/g protein, implying a possible reduction in peptides with aromatic or imidazole side chains or their structural deterioration over time.

**Fig. 4:**
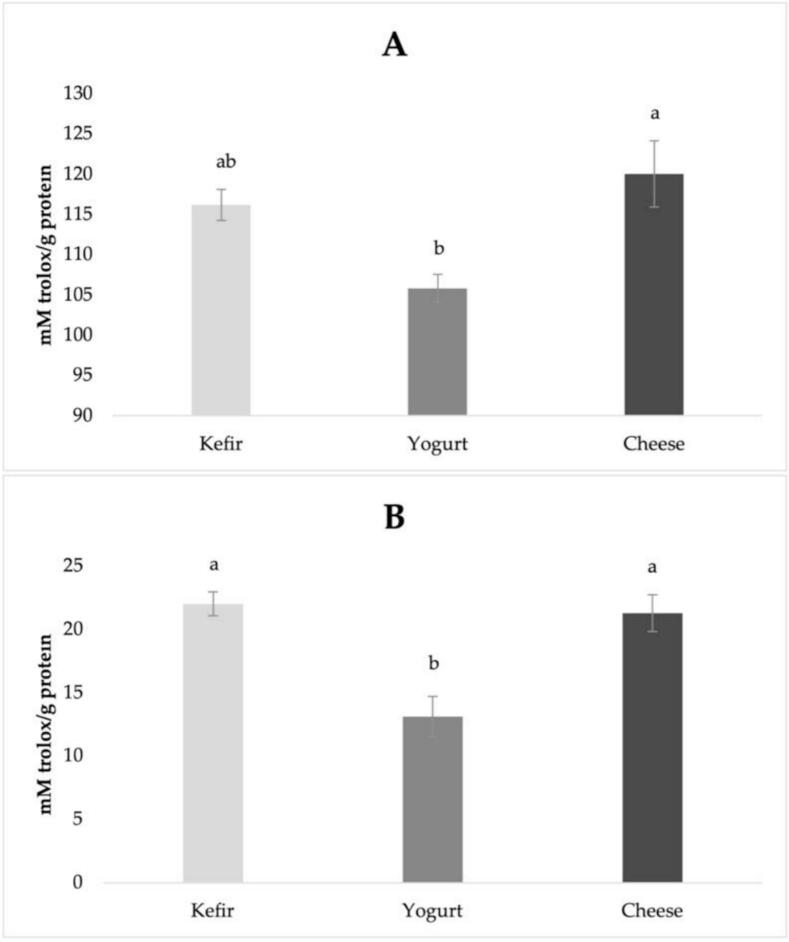
Antioxidant activities of the post-pancreatic <3 kDa permeate of *in vitro* digested kefir yogurt and cheese. A. CUPRAC and B. FRAP analysis. Statistically significant (*P* ≤ 0.05) differences among the mean values were indicated by different letters.

Fig. 4 shows the antioxidant activity of the <3 kDa peptide fractions obtained after protein digestion. A significant portion of these peptides are bioactive peptides produced by or resulting from the action of microorganisms (Aguilar-Toalá et al., 2017). These small peptides play a critical role in the bioavailability of functional foods due to their antioxidant properties (Zhu, Lao, Pan, & Wu, 2022). *D. hansenii* and various *Lactococcus* and *Streptococcus* species were used in kefir; *L.delbrueckii* ssp. *bulgaricus* and *S. thermophilus* were used in yogurt; and *Lc. lactis* ssp. *lactis* and *cremoris* were used in cheese. The protease and peptidase activities of these cultures result in different peptide profiles and various antioxidant capacity.

The <3 kDa peptide fraction of kefir exhibited strong antioxidant capacity, with a CUPRAC value of 116.21 mM Trolox/g protein. This reduction may be attributed to the weakening of the reducing (antioxidant) capacity of certain small peptides generated through protein hydrolysis over time. Conversely, FRAP value was 22.03 mM Trolox/g protein. It may be related to the formation of new antioxidant peptides rich in aromatic and histidine residues. The synergistic activity between yeasts and various lactic acid bacteria in kefir likely contributes to the generation of functionally active and unique peptides, particularly those with antioxidant potential. The opposing trends observed in CUPRAC and FRAP assays reflect the dynamic nature of the antioxidant peptide profile and the structural transformations occurring throughout storage.

The peptides present in the <3 kDa fraction of yogurt exhibited lower overall antioxidant capacity compared to those from kefir and cheese. The <3 kDa peptide fraction of yogurt exhibited the lowest initial CUPRAC value among the samples (105.85 mM Trolox/g protein). While they demonstrated moderate activity in the CUPRAC assay, their performance in the FRAP assay was significantly weaker (13.12 mM Trolox/g protein). The results indicate that the antioxidant potential of small peptides in yogurt appears to be comparatively lower. This may be related to the limited proteolytic activity and lower abundance of peptides capable of participating in electron transfer mechanisms. The use of only *S. thermophilus* and *L.* *delbrueckii* ssp. *bulgaricus* as starter cultures may have limited the extent of proteolysis and peptide diversity.

The <3 kDa peptide fraction of the cheese sample demonstrated a markedly high CUPRAC value (120.07 mM Trolox/g protein) along with a substantial FRAP value (21.31 mM Trolox/g protein), indicating potent antioxidant potential. Among these products, cheese showed the greatest CUPRAC activity and a FRAP value similar to kefir. These findings suggest that cheese is a particularly rich source of low molecular weight peptides with antioxidant function. The higher antioxidant activity may be attributed to whey separation during cheese production, which removes water soluble proteins and concentrates casein-derived components. As a result, the cheese contains a higher concentration of casein derived peptides, likely contributing to its strong antioxidant properties.

## 4 Conclusion

This study demonstrated that fermented goat milk products differ in physicochemical properties, proteolytic patterns, and antioxidant activities depending on product-specific processing conditions and starter cultures. The progressive increase in WSN/TN, TCA-SN/TN, and TFAA values during storage indicates ongoing proteolysis in all products, with the highest extent observed in cheese, followed by kefir and then yogurt. This can be attributed to the dense protein matrix and enzymatic contributions from both rennet and starter culture proteases. Kefir showed considerable antioxidant potential, likely influenced by its complex microbiota, comprising both bacteria and yeast species, while yogurt maintained a relatively lower but stable antioxidant profile during storage. According to the DH analyses, *in vitro* digestion equalized the proteolysis levels across the samples to a comparable extent. The fact that low-molecular-weight (<3 kDa) peptide fractions exhibited antioxidant activity similar to that of post-pancreatic samples suggests that these fractions play a significant role in the overall antioxidant capacity of the products. These results indicate that, beyond fermentation conditions and the selection of starter cultures, protein hydrolysis during digestion and the formation of bioactive peptides strongly affect the antioxidant properties of goat milk products. Overall, kefir, yogurt, and cheese can be regarded as natural sources of antioxidant peptides, supporting their potential roles in the development of functional dairy products. Further research should focus on identifying specific peptide sequences and evaluating their *in vivo* physiological effects.

## CRediT authorship contribution statement

**Hasan Uzkuç:** Writing – original draft, Project administration, Funding acquisition, Formal analysis, Conceptualization. **Sümeyye Sarıtaş:** Writing – original draft, Visualization, Formal analysis. **Nesrin Merve Çelebi Uzkuç:** Writing – original draft, Visualization, Software, Methodology. **Yonca Karagül Yüceer:** Writing – review & editing, Conceptualization. **Tuba Esatbeyoglu:** Writing – review & editing, Supervision, Funding acquisition, Conceptualization.

## Ethical statement

This research involved only chemical analysis of food samples and did not include any human or animal subjects; hence, ethical approval was not applicable.

## Funding

This work is supported by the Scientific Research Coordination Unit of 10.13039/100009055Çanakkale Onsekiz Mart University with grant number: FKB-2024-4788. The publication of this article was funded by the Open Access Fund of the Leibniz Universität Hannover, Germany.

## Declaration of Competing Interest

The authors declare the following financial interests/personal relationships which may be considered as potential competing interests: Given her role as Editor, Tuba Esatbeyoglu, had no involvement in the peer-review of this article and has no access to information regarding its peer-review.

## Data Availability

Data will be made available on request.
